# Characterization of a *Mycobacterium avium* subsp. *avium* Operon Associated with Virulence and Drug Detoxification

**DOI:** 10.1155/2014/809585

**Published:** 2014-05-20

**Authors:** Mariana Noelia Viale, Kun Taek Park, Belén Imperiale, Andrea Karina Gioffre, María Alejandra Colombatti Olivieri, Roberto Damián Moyano, Nora Morcillo, María de la Paz Santangelo, William Davis, María Isabel Romano

**Affiliations:** ^1^Instituto de Biotecnología, Instituto Nacional de Tecnología Agropecuaria, Hurlingham, Buenos Aires 1686, Argentina; ^2^Department of Veterinary Microbiology and Pathology, College of Veterinary Medicine, Washington State University, Pullman, WA 99164, USA; ^3^Laboratorio de Referencia en el Control de la Tuberculosis, Hospital Dr. Antonio A. Cetrángolo, Vicente López, Buenos Aires 8185, Argentina

## Abstract

The *lpr*G-*p55* operon of *Mycobacterium tuberculosis* and *Mycobacterium bovis* is involved in the transport of toxic compounds. P55 is an efflux pump that provides resistance to several drugs, while LprG is a lipoprotein that modulates the host's immune response against mycobacteria. The knockout mutation of this operon severely reduces the replication of both mycobacterial species during infection in mice and increases susceptibility to toxic compounds. In order to gain insight into the function of LprG in the *Mycobacterium avium* complex, in this study, we assayed the effect of the deletion of *lpr*G gene in the D4ER strain of *Mycobacterium avium* subsp. *avium*. The replacement of *lpr*G gene with a hygromycin cassette caused a polar effect on the expression of *p55*. Also, a twofold decrease in ethidium bromide susceptibility was observed and the resistance to the antibiotics rifampicin, amikacin, linezolid, and rifabutin was impaired in the mutant strain. In addition, the mutation decreased the virulence of the bacteria in macrophages *in vitro* and in a mice model *in vivo*. These findings clearly indicate that functional LprG and P55 are necessary for the correct transport of toxic compounds and for the survival of MAA *in vitro* and *in vivo*.

## 1. Introduction

The* Mycobacterium avium* complex (MAC) includes nine species of slow-growing mycobacteria.* Mycobacterium avium* is further classified into four subspecies:* Mycobacterium avium* subsp.* avium* (MAA),* Mycobacterium avium* subsp.* silvaticum* (MAS),* Mycobacterium avium* subsp.* paratuberculosis* (MAP), and* Mycobacterium avium* subsp.* hominissuis* (MAH) [1]. Despite their close taxonomic relationship, the* M. avium* subspecies represent phenotypically diverse organisms with specific pathogenicity and host range characteristics.

LprG is an antigenic lipoprotein with a potential role in bacterial cell wall assembly. This protein was previously described as P27 in the* Mycobacterium tuberculosis* complex [2–5] and the* lpr*G gene has been annotated as* Rv1411c* in the TubercuList web server (http://genolist.pasteur.fr/TubercuList/). LprG induces a strong T helper 1 (Th1) response and simultaneously facilitates the intracellular pathogen multiplication, exacerbating the course of infection with* M. tuberculosis*. Several researches have directed their efforts to find out the mechanism of this paradoxical effect of LprG. For instance, Hovav and collaborators [6] evaluated the effect of LprG in mice inoculated with BCG and demonstrated that an increase in the susceptibility to infection with* M. tuberculosis* in these BCG-inoculated mice overrides the protective effect of the vaccine. Furthermore, Gehring and collaborators [7] showed that LprG is a toll-like receptor 2 (TLR2) ligand that inhibits the processing of major histocompatibility complex (MHC) class II antigens by macrophages. This inhibition may be an attractive mechanism of immune evasion used by* M. tuberculosis*. In addition, the glycolipid binding function of LprG enhances the recognition of bacterial triacylated glycolipids by TLR2 [3]. In MAP, there is a protein of 22 KDa that is recognized by anti-P27 sera that shares 75% identity with its orthologous protein, LprG, in* Mycobacterium leprae* and* M. tuberculosis* complex and elicits a strong cellular and humoral response [8]. Downstream of the LprG-encoding gene, there is an open reading frame (ORF),* Rv1410c*, which encodes a 55 kDa protein (P55) highly similar to the major facilitator superfamily (MFS) membrane proteins from the* Streptomyces* species and other bacteria [9, 10]. P55 was annotated as an aminoglycosides/tetracycline-transport integral membrane protein and functions as an efflux pump that provides resistance to several drugs, probably through a process coupled to the oxidative balance within the cell [9, 10]. P55 of the* M. tuberculosis* complex shares 81% identity with its orthologous in MAA and MAP.

The genomic organization of* lpr*G and* p55* in an operon in* M. tuberculosis* and* M. bovis* [3] suggests that the function of the two proteins is related to each other. The* lpr*G and* p55* genes are conserved across several pathogenic and nonpathogenic* Mycobacterium* species, including* Mycobacterium bovis*,* M. tuberculosis*,* M. avium* subspecies,* M. leprae,* and* Mycobacterium smegmatis*. The knockout of the* lpr*G-*p55* operon causes strong attenuation in* M. tuberculosis* and* M. bovis* when assayed in mice and macrophages [4, 11]. In addition, mutants lacking the expression of the* lpr*G-*p55* operon in* M. tuberculosis* and* M. smegmatis* are more susceptible to toxic compounds, such as ethidium bromide [12, 13]. The multidrug resistance pump inhibitor reserpine inhibits the intrinsic resistance to ethidium bromide in wild type strains of* M. tuberculosis* and* M. smegmatis*, suggesting that* lpr*G-*p55* functions through an efflux-pump mechanism [12, 13].* M. tuberculosis* mutant strains have been shown to be hypersensitive to malachite green and to decolorize this antimicrobial dye faster than the wild type strain. Also,* M. tuberculosis* mutant strains are more susceptible to isoniazid and ethambutol (first-line antituberculosis drugs) as well as to sodium dodecyl sulfate (SDS) [13–15]. In* M. smegmatis*, the cells lacking the* lpr*G-*p55* operon display abnormal colony morphology and are defective for sliding motility [12]. Altogether, these results suggest that the absence of both proteins causes alterations in the cell wall permeability and in its composition and that LprG and P55 act cooperatively in processes that involve the preservation of the cell wall and the transport of toxic compounds in* M*.* tuberculosis* and* M. smegmatis* [12, 13].

In summary,* lpr*G and* p55* are implicated in the resistance to drugs and are encoded in an operon that is important for the virulence of* M. tuberculosis* complex members. In MAC, however, the implication of the presence of these genes is still unknown. The aim of this study was to assess the effect of the* lpr*G gene knockout in the virulence and susceptibility to toxic compounds in MAA.

## 2. Materials and Methods

### 2.1. Gene Nomenclature and Homology

The* MAV_3367* and* MAV_3369* genes in the genome sequence of the MAA strain 104 (GenBank accession number NC_008595) are the orthologous genes to* lpr*G and* p55*, respectively, of* M. tuberculosis* complex. The LprG-encoding gene of MAA (Gene ID: 4529349) has 73% identity with that of* M. tuberculosis*. The MAP strain K-10 (GenBank accession number NC_002944) also has a homologous gene annotated as* lpr*G or* MAP1138c* (Gene ID: 2718014), which has 99% identity to the sequence of the MAA gene. The* p55* gene of MAA (Gene ID: 4525521), which encodes an aminoglycosides/tetracycline-transport integral membrane protein, has 90% homology to* p55* of* M. tuberculosis*. In MAP,* p55* is annotated as* MAP1137c* (Gene ID: 2718013) and is 99% homologous to the sequence of the MAA* p55* gene.

### 2.2. Bacterial Strains and Culture Media

*Escherichia coli* strains were grown either in Luria-Bertani (LB) broth or on LB agar supplemented with the corresponding antibiotics.* M. smegmatis* strains were maintained in Middlebrook 7H9 medium supplemented with AD (0.5% bovine serum albumin, 0.2% glucose).

The avian purified protein derivative (PPDA) is prepared from the D4ER strain of MAA and is used as the basis of the double comparative delayed-type hypersensitivity (DTH) skin test in Argentina [16, 17]. For this reason, we selected this MAA strain to develop a mutant. MAA was grown in Middlebrook 7H9 medium supplemented with AD, 0.5% glycerol, and 0.05% Tween 80 to avoid clumps. Solid cultures where plated on Middlebrook 7H10 medium supplemented with AD and 0.5% glycerol. When necessary, hygromycin (Hyg) was added to the medium at a concentration of  75 *μ*g/mL. All cultures were routinely incubated at 37°C in a shaking incubator (100 rpm).

### 2.3. Generation of* lpr*G Mutant of MAA

**Figure 1:**
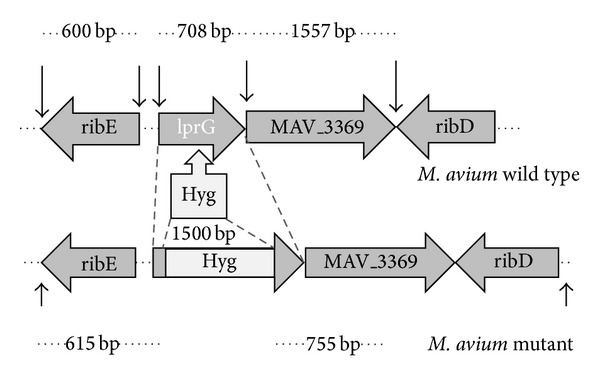
Mutation of the* lprG* gene of* Mycobacterium avium* strain D4ER. Genomic region with flanking genes of* lprG* gene and insertion of hygromycin (Hyg) resistant cassette is indicated. Arrows above represent the length of* lprG* and flanking genes in* M. avium* (sequenced strain 104). Arrows at the bottom indicate expected bands after digestion of* M. avium lprG* mutant with restriction enzymes. Values between arrows indicate the molecular weight of genes or expected bands, expressed in base pairs (bp).

The* lpr*G deletion mutant of* MAA* was generated by allelic exchange mutagenesis using the specialized transduction system described by Bardarov et al. [18]. Briefly, the primers* lpr*Gup-Forward (*Bgl* II) and* lpr*Gup-Reverse (*Hind* III) (Table 1 and Figure 1) were used to amplify the* lpr*Gup region, while the primers* lpr*Gdown-Forward (*Xba* I) and* lpr*Gdown-Reverse (*Afl* II) (Table 1 and Figure 1) were used to amplify the* lpr*Gdown region. The* lpr*Gup region corresponds to the first 48-bp of* lpr*G coding region plus the 567-bp-flanking 5′ upstream region. The* lpr*Gdown region consists of the genomic region containing a 250-bp end in the downstream region of the* lpr*G gene plus 505-bp-flanking 3′ region. All amplifications were performed by PCR.

The fragments* lpr*Gup and* lpr*Gdown containing restriction sites were directionally cloned into pYUB854 on either side of the Hyg resistance (Hyg^r^) gene to generate the allelic exchange substrates (AESs) (Figure 1). The pYUB854 containing AESs (pYUB854-*lpr*G) were packed into the phasmid phAE87 using an* in vitro* packaging solution (Gigapack III; Stratagene) in* E. coli* HB101 and plated on LB agar with Hyg. The recombinant phasmid, phAE87 with AESs, was prepared from the pooled Hyg-resistant colonies and electroporated into* M. smegmatis* mc^2^ 155 to generate a transducing mycobacteriophage. After electroporation, top agar (0.6%) was added to the bacteria and the mix was plated on Middlebrook 7H10 medium and incubated at the permissive temperature (30°C) for 3 days to generate phage lysates. All transducing phages were plaque-purified and tested to confirm the phenotype by PCR with locus specific primers. Finally, the high titer transducing mycobacteriophages were prepared (10^10^ plaque forming units PFU/mL) and mixed with an equal volume of a MAA culture with an optical density (OD) at 600 nm of 0.6, which had been previously centrifuged and resuspended in MP buffer (50 mM Tris-HCl pH 7.6, 150 mM NaCl, 10 mM MgCl_2_, 2 mM CaCl_2_). After the recovery time, the culture was plated on 7H10 agar with 75 *μ*g/mL Hyg at 37°C for 2 weeks and colonies were finally selected for analysis.

### 2.4. Confirmation of Allelic Exchange Mutants: DNA Extraction and PCR

After 2 weeks of incubation on selective agar, each Hyg^r^ colony was subcultured on a new selective agar plate with Hyg to expand bacterial cultures for subsequent analyses. DNA from the mycobacterial strains, wild type, and candidate mutant colonies were extracted by the CTAB method as described previously [19] and the gene disruption was confirmed by PCR. Each PCR was performed with specific primer pairs for amplifying* lpr*G and IS*1245*. IS*1245* is a specific insertion element from* M. avium* that allows the identification of strains within this species. In this study, IS*1245* was amplified with primers designed by Guerrero et al. [20] (Primers IS1245 Forward and Reverse Table 1), while* lpr*G was amplified with primers* lpr*G-0 Forward and Reverse (Table 1).

### 2.5. RNA Isolation and RT-PCR

The pellet from a 150-mL culture of the wild type MAA and the mutant colony selected (MAAΔ*lpr*G) were resuspended in 1 mL Trizol reagent (Sigma) and transferred to 2-mL screw-cap microcentrifuge tubes containing 0.1-mm-diameter zirconium beads (Bio 101) for disrupting the cells. The disruption was performed at 6.5 speed (4 cycles of 30 s each, cooling between cycles). The aqueous phase was subsequently extracted twice with chloroform and precipitated with isopropanol. The remaining DNA in the samples was digested with DNAse I (Invitrogen) for 15 min at room temperature, followed by DNAse I inactivation at 65°C for 5 min. The RNA samples were repurified by using an RNA purification kit, according to the manufacturer's instructions (RNeasy Qiagen). The examination of the purified total RNA by 1% agarose gel electrophoresis revealed prominent 23S, 16S, and 5S ribosomal bands. The first strand of cDNA was synthesized using 3 *μ*g of total RNA from the wild type or MAAΔ*lpr*G strains as templates. Random hexamers were used for the reaction. This synthesis was performed following the indications in the SuperScript Preamplification System for First Strand cDNA Synthesis kit (Life Technologies). Ribonuclease-treated RNA samples were used as a negative control. 5 *μ*L aliquot of the cDNA synthesis reaction was amplified with two sets of primers for the* lpr*G (*lpr*G-1 Forward and Reverse and* lpr*G-2 Forward and Reverse) and* p55* (*p55*-1 and* p55*-2 Forward and Reverse) genes, respectively (Table 1). The housekeeping* gapdh* gene was used as a positive control using the corresponding primers (Table 1). The amplification conditions are also indicated in Table 1. The amplification products were detected in 1% agarose gels.

### 2.6. Construction of Complemented MAAΔ*lpr*G Strain

For the complementation of the mutant phenotype, a fragment containing the* lpr*G gene or the complete* lpr*G-*p55* operon was PCR-amplified from the wild type MAA genomic DNA using the primers* lpr*G-compl or operon-compl Forward and Reverse, respectively (Table 1). The PCR products were cloned into pGEM-T vector (Promega) and subcloned into pVV16 to produce the plasmids p*lpr*G or p*lpr*G-*p55*. The shuttle vector pVV16 has an* E. coli* origin of replication, the mycobacterial pAL5000 origin of replication, a gene for kanamicin resistance, and the hsp60 promoter. These plasmids were used to transform MAAΔ*lpr*G by electroporation and, in turn, obtain the strains MAAΔ*lpr*G*::lpr*G and MAAΔ*lpr*G*::lpr*G-*p55*. Colony selection was performed on plates with kanamycin. Complementant strains were confirmed by PCR and western blot.

### 2.7. Protein Extraction and Western Blot

Protein extractions from wild type MAA, MAAΔ*lpr*G, and the complemented strains were done as previously described [2]. Briefly, cell pellets from 50-mL cultures were washed twice using ice cold 10 mM Tris-base (pH 9.5) with a protease inhibitor cocktail. The cells were then resuspended in 1 mL of the same buffer and transferred to 2 mL screw-cap microcentrifuge tubes containing zirconium beads and disrupted by using a ribolyser glass bead as previously described. Western blot assays were performed with mycobacterial proteins fractionated on 12%-SDS polyacrylamide gel electrophoresis and transferred to nitrocellulose membranes (Bio-Rad). The membranes were blocked with 5% nonfat dried milk in Tris-buffered saline (TBS), washed with TBS, and probed with anti-LprG polyclonal antibody in a 1 : 500 dilution. An alkaline phosphatase-conjugated anti-rabbit immunoglobulin G (Sigma) dilution of 1 : 2000 was used as a secondary antibody. BCIP/NBT (5-bromo-4-chloro-3-indolyl phosphate/nitroblue tetrazolium) Color Development (Promega) was used for the colorimetric detection. The protein Hsp70 was used as an internal control.

### 2.8. Drug Susceptibility Testing by the Proportion Method and the Colorimetric-Based Method

The indirect proportion method on Middlebrook 7H11 was used to determine the minimal inhibitory concentration (MIC) of some drugs used in* M. tuberculosis* and* M. avium* complex treatment and therefore compare the drug resistant profiles of wild type MAA strain and MAAΔ*lpr*G [21]. The drugs tested were isoniazid (INH), rifampicin (RIF), kanamycin (KAN), levofloxacin (LX), and p-nitro-benzoic acid (PNB) (19, 20, 21). MICs were assayed over the following concentrations of antibiotics: 0.2 *μ*g/mL INH, 1.0 *μ*g/mL RIF, 6.0 *μ*g/mL KAN, 2.0 *μ*g/mL LX, and 500.0 *μ*g/mL PNB.

A microdilution colorimetric method was used for MIC of other drugs: p-aminosalicylic acid (PAS), ethionamide (ETH), cycloserine (CS), clarithromycin (CLA), azithromycin (AZ), amikacin (AMK), moxifloxacin (MOX), linezolid (LZ), and rifabutin (RBT). Briefly, a noncommercial, microplate colorimetric-based method with resazurin (REMA) was used as a general indicator of cell growth and viability, following a previously described method [21–24]. In this method, a 96-well, microtiter, flat bottom plates were used to perform drug susceptibility tests. Serial twofold dilutions of the drugs were performed and wells were left free of drugs to be used as growth controls. The wells were inoculated with 100 *μ*L of a 1 : 25 bacterial suspension with turbidity comparable to 1.0 MacFarland standard (original bacterial suspension). The plates were incubated at 37°C for 5 days at a normal atmosphere. After the incubation period, 0.02% resazurin was added to the wells and incubated for 24 h. The MIC for each particular drug was considered as the lowest concentration showing less change in color compared to the growth controls. The ranges of drug concentrations used were 8.00–0.25 *μ*g/mL with PAS and cut-off 2.00 *μ*g/mL; 8.00–0.025 *μ*g/mL with ETH and cut-off 2.00 *μ*g/mL; 120.00–3.75 with CS and cut-off 30.00 *μ*g/mL; 8.00–0.25 *μ*g/mL with CLA and cut-off 2.00 *μ*g/mL; 8.00–0.25 *μ*g/mL with AZ and cut-off 2.00 *μ*g/mL; 8.00–0.25 *μ*g/mL with AMK and cut-off 4.00 *μ*g/mL; 4.00–0.13 *μ*g/mL with MOX and cut-off 0.50; 4.00–0.13 *μ*g/mL with LZ and cut-off 0.50; 2.00–0.03 with RBT and cut-off 0.25 *μ*g/mL.

The methods and the concentration ranges for each drug were based on the clinical standard protocols used for* M. tuberculosis* and* M. avium* complex drug susceptibility testing at the Tuberculosis Control Program Reference Laboratory, “Dr. Cetrángolo” Lung disease Hospital, Buenos Aires Province, Argentina.

### 2.9. Ethidium Bromide Sensitivity Assays

To evaluate the susceptibility of wild type MAA and MAAΔ*lpr*G to ethidium bromide, we determined the MIC of each strain to this toxic compound with the colorimetric method previously explained for the antituberculosis drugs. The range of concentration used was 10–0.3 *μ*g/mL.

To analyze the growth kinetic in the presence of ethidium bromide, wild-type MAA and MAAΔ*lpr*G were grown in liquid medium in the presence of 1 *μ*g/mL ethidium bromide. When necessary, reserpine at a concentration of 125 *μ*g/mL was added into the cultures. Reserpine is a plant alkaloid which modulates multidrug efflux pumps that is often used in functional assays [12, 13]. The bacterial growth was monitored by OD and the growth rate was compared to that in the absence of ethidium bromide.

### 2.10. Mouse Infection Studies

BALB/c mice were intranasally (i.n) or intraperitoneal (i.p) inoculated with bacillary suspensions with a known concentration of 1 × 10^7^ viable cells in 50 *μ*L phosphate buffer saline (PBS). Each animal was anesthetized with Ketamine/xylazine and inoculated with wild-type MAA or MAAΔ*lpr*G strains. Four mice per group were sacrificed at 20 and 80 days postinfection (dpi) and the lungs were removed and homogenized (i.n infections). Other four mice per group were sacrificed at 2, 7, 14, and 70 dpi and the spleens were collected from individual animals (i.p infections). The tissues were homogenized in 1 mL ice-cold PBS using a tissue homogenizer and serial dilutions of each homogenate were spread onto duplicate Middlebrook 7H10 agar medium. The number of colony forming units (CFUs) was calculated after 2 to 3 weeks. All experiments were done according to the instructions of the Committee for Institutional Care and Use of Animal Experimentation (CICUAE) of INTA.

### 2.11. Macrophage Infection Studies

The murine macrophage-like cell line J774 was infected as previously described [25], with some modifications. Briefly, J774 cells were resuspended in RPMI-1640 complete medium (with 4 mM L-glutamine, 1 mM/L pyruvate, 4500 mg/L glucose) and 2% fetal bovine serum (FBS) and adjusted to a concentration of 10^5^ cells/mL. The cells were distributed (1 mL per well) into 24-well tissue culture plates and were allowed to adhere for 24 h at 37°C in 5% CO_2_ in a humidified atmosphere. Macrophages were infected with a multiplicity of infection (MOI) of 5 with wild type MAA, MAAΔ*lpr*G, MAAΔ*lpr*G*::lpr*G, and MAAΔ*lpr*G*::lpr*G-*p55* strains. The infected cells were incubated for 3 h (*t* = 0) and then washed three times with warm PBS. At 0, 24, 48, and 72 hours postinfection (hpi), the cells were scraped and lysed with 0.5 mL 0.1% Triton X-100 for 15 min. The lysate was serially diluted in PBS and plated on 7H10 agar to assess the CFUs. This experiment was performed in triplicate.

### 2.12. Statistical Analysis

CFU data were analyzed with Microsoft Excel statistical software using Student's* t*-test. The* P* values < 0.05 were considered statistically significant.

## 3. Results

### 3.1. Generation of* lpr*G-Knockout Strain of MAA

In the present study, we generated the* lpr*G deletion mutant MAAΔ*lpr*G from MAA using a specialized transduction system, which was previously described by Bardarov et al. [18]. PCR with the specific* lpr*G primers was performed to confirm the allelic replacement and absence of* lpr*G in those colonies that were Hyg resistant. One MAAΔ*lpr*G clone was selected among PCR-negative clones for further studies. As an internal control, the identity of the MAA colonies was corroborated by PCR using primers against IS*1245* (data not shown).

**Figure 2:**
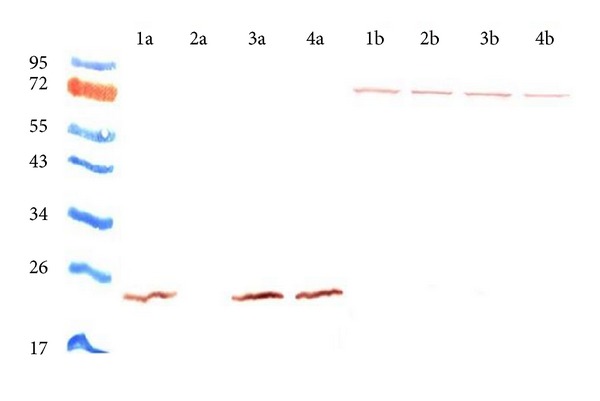
Detection of the protein LprG in MAA wild type (1), MAAΔ*lpr*G (2), MAAΔ*lpr*G::*lpr*G (3), and MAAΔ*lpr*G::*lpr*G-p55 (4). Western blot was performed with anti-LprG sera (a) and anti-Hsp70 sera (b) as internal control. LprG was not detected in the MAAΔ*lpr*G protein fraction, while the complemented strains restored its expression.

To confirm that the chromosomal mutation disrupted* lpr*G synthesis, cell lysates from wild type MAA and MAAΔ*lpr*G were analyzed by SDS-PAGE and western blot using anti-LprG polyclonal mice sera. A 22-KDa band corresponding to the expected size for LprG was observed in whole-cell extracts from the wild type MAA strain but it was absent in MAAΔ*lpr*G. These results confirmed the disruption of the* lpr*G gene and the absence of the protein in the mutant strain (Figure 2). We also included the complemented strains in the western blot analyses, demonstrating that the complementation restored the expression of the protein (Figure 2).

**Figure 3:**
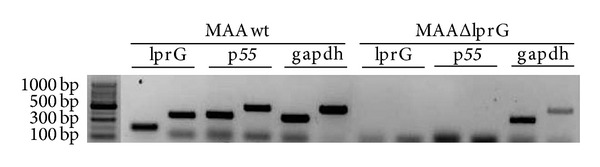
RT-PCR of* lprG*,* p55,* and the housekeeping gene glyceraldehyde-3-phosphate dehydrogenase (*gapdh*) were used as the internal control. MAA wild type and MAAΔ*lprG* cDNA were obtained using random hexamers for conversion of mRNA. Two different specific primers pairs were used to amplify* lprG* (*lpr*G-1 and* lpr*G-2),* p55* (*p55*-1 and* p55*-2), and* gapdh* (*gapdh 1* and* gapdh 2*). The introduction of a Hyg resistance cassette in the coding sequence of* lpr*G of the MAA D4ER strain induced a polar effect on the expression of the downstream gene* p55*.

In a previous study, it has been demonstrated the presence of polycistronic mRNA species containing both* lpr*G and* p55* transcripts in* M. bovis* and* M. tuberculosis* [3]. To investigate the effect of* lpr*G mutation on the expression of* lpr*G and* p55* inMAA, we conducted a RT-PCR. The transcription of* lpr*G and* p55* was evaluated using different pairs of primers for* lpr*G and for* p55* (Table 1). Neither* lpr*G nor* p55* mRNAs were observed in MAAΔ*lpr*G (Figure 3), which indicates that the insertion of the Hyg-resistance cassette in* lpr*G gene exerts a polar effect on* p55*. This result is consistent with the polycistronic arrangement of these genes in* M. tuberculosis* complex.

### 3.2. Role of LprG and P55 from MAA in the Resistance to Antibiotics and Toxic Compounds

Several first- and second-line drugs are used against* M. tuberculosis* and* M. avium* complex. To explore the susceptibility of MAA to some of these drugs in the absence of* lpr*G and* p55*, we tested the resistance of MAAΔ*lpr*G by two different methods based on the protocol standardized at the “Dr. Cetrángolo” Lung disease Hospital, Buenos Aires Province, Argentina. While the lack of LprG and P55 did not affect the resistance to INH, LX, PNB, PAS, ETH, and CS, and also did not affect susceptibility to KAN, CLA, and AZ, MAAΔ*lpr*G was more susceptible to RIF, AMK, LZ, MOX, and RBT than the wild type MAA strain (Table 2).

**Figure 4:**
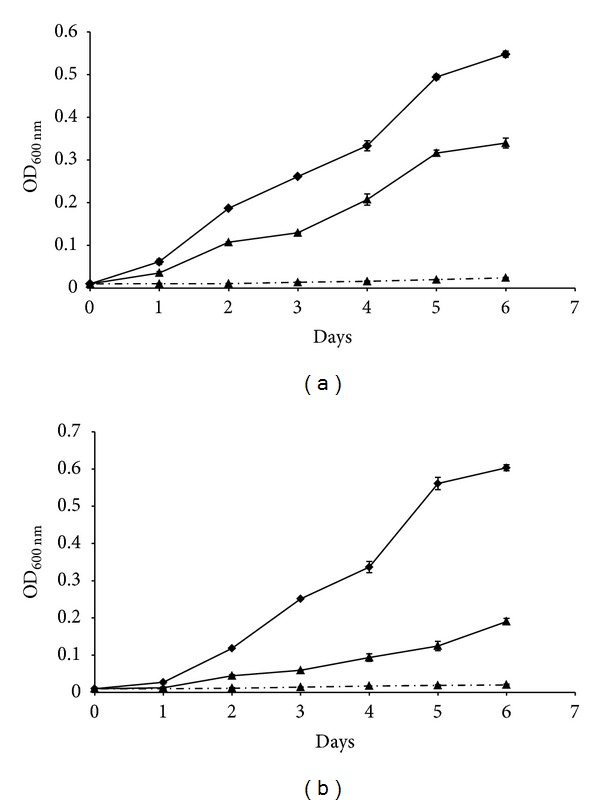
Ethidium bromide susceptibility and effect of pump inhibitor reserpine in MAA wild type (a) and MAAΔ*lpr*G (b). Bacterial strains were grown with 1 *μ*g/mL of ethidium bromide (full line with triangles), without ethidium bromide (full line with diamonds) or with 1 *μ*g/mL of ethidium bromide plus reserpine (dotted lines with squares).

Taking into account that the deletion of the* lpr*G-*p*55 operon in* M. smegmatis* [12] and* M. tuberculosis* [13] makes these bacteria more susceptible to ethidium bromide, we decided to assay the susceptibility of MAA to this toxic compound. We first determine the MIC of ethidium bromide for the wild type MAA, MAAΔ*lpr*G, and both complemented strains by the colorimetric method with resazurin. Wild type MAA showed a MIC of 5, while the MAAΔ*lpr*G strain exhibited a MIC of 2.5, indicating a higher susceptibility of the mutant to the toxic compound. We then assayed the MIC for the complemented strains. MAAΔ*lpr*G*::lpr*G showed a MIC of 2.5 and MAAΔ*lpr*G*::lpr*G-*p55* showed a MIC of 5, similar to those obtained for MAAΔ*lpr*G and wild type strains, respectively. These results suggest that P55 is implicated in the resistance to this toxic compound in MAA. To assess efflux of ethidium bromide, we chose a concentration that ensures that its accumulation is lower than that which causes ethidium bromide to reach and intercalate into DNA (lower than half the MIC for the mutant strain). The MAAΔ*lpr*G strain showed higher sensitivity to 1 *μ*g/mL of ethidium bromide (Figure 4(b)) than the wild type MAA strain (Figure 4(a)). The presence of the pump inhibitor reserpine reversed the intrinsic resistance of the wild type strain to ethidium bromide (Figure 4) indicating that resistance to this toxic compound is caused by a mechanism dependent on efflux pumps.

### 3.3. *In Vivo* Growth Studies

**Figure 5:**
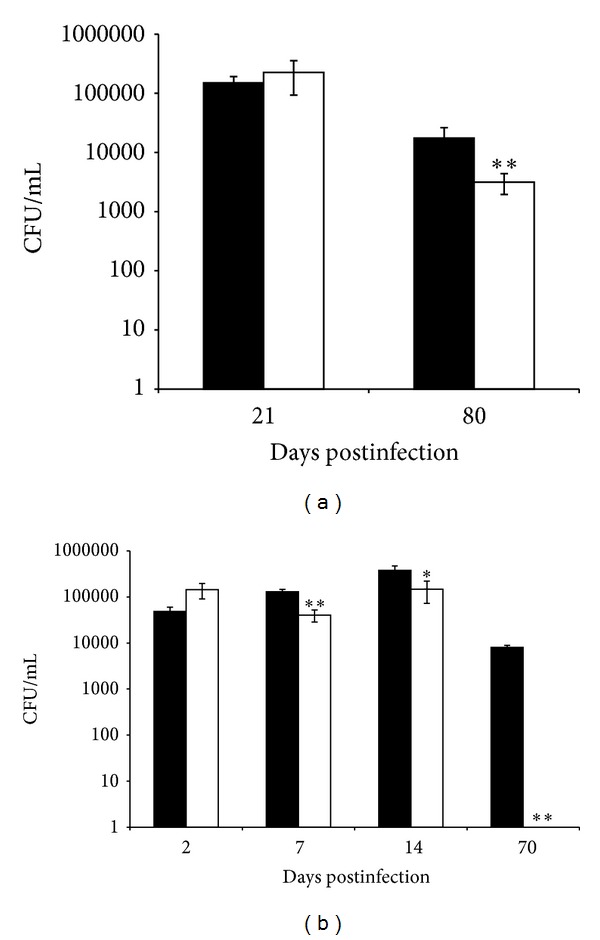
Mouse infections with MAA wild type (black bars) and MAAΔ*lprG* (white bars). BALB/c mice were intranasally (a) or intraperitoneally (b) inoculated with bacillary suspensions to 1 × 10^7^ viable cells. Four mice per group were sacrificed at 21 and 80 (a) or 2, 7, 14, and 70 (b) days postinfection and lungs (a) or spleen (b) were removed. The CFUs were determined by four dilutions of each homogenate spread onto duplicate plates. The values are expressed as the means ± SD of log CFU in four mice. These data are based on two independent experiments with similar results. Significantly different from values of the wild type strain **P < 0.01, *P < 0.05.

The infection and survival patterns of MAAΔ*lpr*G were evaluated* in vivo* to examine the role of LprG and P55 in the virulence of MAA. We used the intranasal and intraperitoneal route to infect BALB/c mice and determined organ colonization in the lung and spleen by colony forming units (CFUs) counting at different time points. With the intranasal infection, the CFUs in the lungs of both strains were equivalent to 21 dpi, and these values were 10 log higher than the values obtained at 80 dpi. However, at this later time point, the burden of MAAΔ*lpr*G in the lungs exhibited a significant reduction compared to that of the wild-type MAA strain (Figure 5(a)).

With the intraperitoneal inoculation, a significant reduction in the number of CFUs was detected at 7, 14, and 70 dpi in the spleens of mice inoculated with MAAΔ*lpr*G compared to the mice inoculated with wild-type MAA. Very few or no bacteria were found in the spleens 70 dpi with MAAΔ*lpr*G (Figure 5(b)).

### 3.4. Macrophage Infection

**Figure 6:**
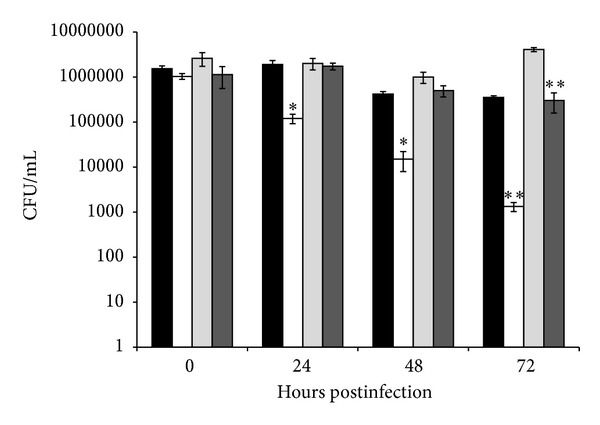
Bacterial growth in J774 macrophages assessed by CFU at 0, 24, 48, and 72 hours postinfection. Infection with MAA wild type (black bars), MAAΔ*lpr*G (white bars), MAAΔ*lprG*::*lpr*G (light grey bars), and MAAΔ*lpr*G::*lpr*G-*p55* (strong grey bars) performed at a MOI of 5. Data indicate the mean ± SD of log CFU by triplicate. Significantly different from values of the wild type strain **P < 0.01, *P < 0.05.

Next, we used the murine macrophage cell line J774 to determine whether the attenuated phenotype of MAAΔ*lpr*G observed in mice was a consequence of an inability of the MAAΔ*lpr*G strain to subvert the macrophage microbicidal mechanism. For this purpose, we assessed the intracellular survival of wild-type MAA and MAAΔ*lpr*G. We also assayed the virulence of complemented strains MAAΔ*lpr*G*::lpr*G and MAAΔ*lpr*G*::lpr*G-*p55 in vitro* in the murine macrophage. CFUs of MAAΔ*lpr*G were significantly lower than that of the wild-type and complemented strains (Figure 6) and the replication rate of MAAΔ*lpr*G was steady and low. These results indicate that the mutation of* lpr*G impaired the replication of MAA inside murine macrophages. Furthermore, we demonstrated that the wild-type phenotype is restored with the whole operon complementation; which indicates that LprG or P55 or both are necessary for establishing a successful infection, replicating and persisting in host cells. Unexpectedly, complementation with only the* lpr*G gene significantly increased the replication in macrophages after 72 hpi. Since LprG is implicated in the virulence of bacteria of* M. tuberculosis* complex, this result confirms a similar role in MAA.

## 4. Discussion

LprG is a surface-expressed lipoprotein which is also secreted. This protein has been implicated in the modulation of the host immune response during* M. tuberculosis* infection, inhibiting the antigen presenting cells to process and present MHC-II antigens [7]. This antigenic membrane protein in* M. tuberculosis* and* M. bovis* was first identified by Bigi and collaborators in 1997 [2] and named P27 because of its molecular weight. The* lpr*G gene is part of an operon in* M. tuberculosis*,* M. bovis,* and* M. smegmatis*, cotranscribed with its adjacent gene* p55* (Rv1410c) [3, 12].

In this study, we demonstrated that the introduction of a Hyg resistance cassette in the coding sequence of* lpr*G of the D4ER strain of MAA induced a polar effect on the expression of the downstream gene* p55*. This result and the fact that there is a close proximity between* lpr*G and* p55* in the same transcriptional orientation suggest that both genes are also part of an operon in* M. avium*. However, there is a possibility that the downstream gene* p55* could have a promoter for itself in the fragment of* lpr*G replaced with the Hyg resistance, so we cannot exclude marginal expression of* p55* in conditions different to those that we have analysed. Also, operons with more than one promoter have been described in mycobacteria which are active under different conditions [26–28]. Regardless of the function of the individual proteins, the putative organization in an operon could suggest that the two protein functions are related both in the* M. tuberculosis complex* and in the* M. avium complex*. In the present study, the significantly impaired growth and persistence of MAAΔ*lpr*G in macrophages and in a mice model suggest that the expression of LprG or P55 or both is involved in the bacterial virulence. Bacterial attenuation in the mutant strain makes it susceptible to the bactericide mechanisms of phagocytic cells. However, the conservation of the operon in the nonpathogenic mycobacteria* M. smegmatis* suggests that the biological role of these proteins is also required during environmental growth. Rv1410c/*p55* gene encoding the P55 efflux pump of the* M. tuberculosis* complex has been postulated to be involved in drug resistance because of its homology with the major facilitator superfamily (MFS) antibiotic transporters from other bacteria [29, 30] and is annotated as an aminoglycosides/tetracycline-transport integral membrane protein. This transporter belongs to the drug/H^+^ antiporter-14-transmembrane domain (DHA14) family, whose members are thought to export cationic small molecules by proton motive force; this is the main source of energy used by the P55 efflux pump to extrude drugs [12]. Characterized members of the DHA14 transporter family were identified based on their ability to confer drug resistance when heterologous P55 from* M. tuberculosis* and* M. bovis* was expressed in* M. smegmatis* [12]. P55 from* M. tuberculosis *confers resistance to amynoglycosides [12]. In accordance with these findings, in the present study we demonstrated that a mutant with loss of LprG and P55 in MAA has a reduced resistance to rifampicin, amikacin, moxifloxacin, linezolid, and rifabutin. Ramón-García and collaborators [10] proposed that P55 plays an essential role as an efflux pump in detoxification processes coupled to oxidative balance within* M. tuberculosis*.* M. tuberculosis* possesses many efflux pumps and their roles in drug resistance and physiology are actively investigated. Efflux pumps are also involved in drug tolerance because their increased expression may allow bacteria to survive otherwise lethal concentrations of substrate antibiotic and thus allow selection for target mutations that increase drug resistance levels [31, 32]. Therefore, efflux pumps extrude a wide variety of chemically unrelated compounds conferring multidrug resistance and participate in numerous physiological processes. The role of these pumps in drug resistance should be actively investigated in different mycobacteria to develop pump inhibitors that could provide valuable tools against mycobacterial diseases. The high susceptibility of MAAΔ*lpr*G to ethidium bromide and the finding that reserpine, a pump inhibitor, alters the intrinsic resistance of MAA parental strain to ethidium bromide indicate that the susceptibility observed in MAAΔ*lpr*G is due to the lack of the efflux pump function. The MIC of ethidium bromide found in the mutant strain is lower than the MIC of the wild type MAA, further confirming these results. MAAΔ*lpr*G*::lpr*G shown the same MIC that MAAΔ*lpr*G, while MAAΔ*lpr*G*::lpr*G-*p55* displayed a MIC similar to the wild type strain. The findings support the fact that P55 or both proteins (P55 and LprG) are necessary for the proper operon activity and that P55 may be implicated in efflux transport in* M. avium* complex. Therefore, this operon probably contributes to the resistance to environmental toxins in MAA, as demonstrated in* M. tuberculosis* [13]. The ability of P55 to transport substrates is likely to be crucial to its physiological role in* M. tuberculosis* during infection [11] and, according to our results, also for MAA.

*M. tuberculosis* lipoproteins are major antigens and trigger the activation of cellular and humoral immune responses to mycobacteria. Lipoproteins are potent agonists of TLR2, which upon long term stimulation has been associated with the downregulation or deviation of the immune response. Within the* M. tuberculosis* genome, the lipoproteins LprG and LppX share significant homology. LppX is required for the translocation of the cell wall lipid phthiocerol dimycocerosate (PDIM) and functionally associated with the resistance-nodulation-cell division small molecule transporter Mmpl7, which exports PDIM across the cell membrane. The residues in LppX that are shared by LprG constitute the pocket within which PDIM is thought to reside [12]. Given the structural homology between LppX and LprG, we hypothesized that LprG could contribute to the transport of a lipid substrate to P55. In addition, LprG binds other glycolipids as lipoarabinomannan (LAM), lipomannan (LM), and phosphatidylinositol mannosides (PIM), suggesting that it may be a carrier of these important components of the bacterial cell wall [33–35]. Therefore, the importance of LprG in virulence could be due to its contribution to assembly, trafficking, and cell wall insertion of glycolipids. These findings led us to hypothesize that a mutant in the* lpr*G gene could be used as a vaccine against mycobacterial diseases.

It was previously demonstrated that the integrity of the* lpr*G-*p55* operon is crucial for replication of* M. tuberculosis* and* M. bovis* [5, 11]. In this study, we demonstrated that the knockout mutation of the* lpr*G also impaired the replication and persistence of MAA in macrophages. It is worthy to mention that the macrophages assays represent the behavior of the* M. avium* strains during the early stage of infection; then we hypothesized that MAA mutation prevents correct cell wall insertion of glycolipids affecting its virulence and replication within macrophages. In order to confirm that the phenotype observed was due to the absence of LprG and P55, we restored a copy of the wild type genes. We transformed MAAΔ*lpr*G with an expression plasmid of mycobacteria containing the whole operon or the* lpr*G gene only and we evaluated the virulence in macrophages. The complementation with the whole operon restored the wild type phenotype, indicating that this operon was necessary for a successful infection and replication in the host cells. These results are comparable with that observed by Bianco and collaborators in* M. bovis* [7], where a mutant strain in* lpr*G-*p55* restored the wild type phenotype in murine macrophages when it was complemented with the whole operon. Unexpectedly, MAAΔ*lpr*G*::lpr*G increased significantly the replication in macrophages at 72 dpi. This effect could be attributed to the estequiometric balance in the expression of LprG because of the overexpression under a strong promoter. The overexpression could change the regulation of endogen mechanisms, suggesting an infection facilitator effect of the protein as it was described in* M. tuberculosis* complex [6]. The inoculation of LprG in mice produced an increase in the susceptibility to the infection with* M. tuberculosis* and the coinoculation of LprG with BCG annulated the protective effect of the vaccine [6]. Our results suggest that the protein LprG in MAA could exacerbate bacteria proliferation, beating the immunosuppressant effect of macrophages.

In conclusion, although the exact function of LprG and P55 of MAA should continue under study, the evidence that restitution in MAA mutant of only LprG was not enough to return to wild type resistance to toxic compounds supports the hypothesis that P55 is involved in transport processes in MAA. However, if LprG and P55 are encoded in the same operon, it suggests that the two protein functions are related. LprG seems to be necessary for the transport of toxic compounds across the bacterial cell wall and this transport in turn may be mediated by P55. The reason for the multidrug-resistant (MDR) phenotype of MAA is its mycobacterial cell envelope, which is relatively impermeable to antibiotics. Efflux pumps can play an important role in their antibiotic resistance preventing toxic compounds from reaching their targets. Furthermore, LprG and P55 are likely to function in cooperation in MAA to counterattack the microbicidal action of macrophages, as demonstrated in* M. tuberculosis*.

Our findings will help to understand efflux-mediated drug resistance in mycobacteria belonging to the MAC and will contribute to the knowledge for future development of effective antimycobacterial therapies.

## Figures and Tables

**Table 1:** Primer sequences, annealing temperatures (*T_a_*), and sizes of amplified products.

Name	Primer sequence Forward	Primer sequence Reverse	*T_a_* (°C)	Size (bp)
*lpr*Gup	*ctgagatctggacaccgtcagcgagatgc *	*gtcaagcttgagggtcagggatgcgaaaa *	63	613
*lpr*Gdown	*gtctctagaggccgcgaaaccatcaac *	*tgtcttaagcgccagtcgtggaacaggaag *	63	754
*lpr*G-0	*tcctggacccacacggtgct *	*gggctccaagcagagcggtg *	60	510
*IS1245 *	*gccgccgaaacgatctac *	*aggtggcgtcgaggaagac *	62	427
*lpr*G-1	*ctatcggccgttttcgcatc *	*acgctcttgacgttcttggt *	58	146
*lpr*G-2	*ctgagcatccagggcaagat *	*ctgaccgttgatggtttcgc *	58	309
*p55*-1	*cgatgtacctgctgggctac *	*atgaagatcccgtacagcgg *	58	325
*p55*-2	*gtgttctggatcaacgtgcc *	*gcgatcaagaaccacagcag *	58	443
*gapdh 1 *	*ccgtcgtacttgtcgtcgtt *	*ggctcacctgctgaaattcg *	58	306
*gapdh 2 *	*cgcgtcgtagtacttcagga *	*cgtcaacgacgacaagtacg *	58	443
*lpr*G-compl	*catatgatgcagacccgccgc *	*catatgtcacgagctcaccgg *	61	708
operon-compl	*catatgatgcagacccgccgc *	*catatgctaacgctcacccagcg *	62	2,265

**Table 2:** Drug susceptibility of MAA wild type and MAAΔ*lpr*G.

Drug	*M. avium* wild type	*M. avium *Δ*lpr*G
INH^a^	Resistant	Resistant
RIF^a^	Resistant	Susceptible^c^
LX^a^	Resistant	Resistant
KAN^a^	Susceptible	Susceptible
PNB^a^	Resistant	Resistant

Determination of MIC (*μ*g/mL)		
PAS^b^	**>8.0 **(resistant)	**>8.0 **(resistant)
ETH^b^	**>8.0 **(resistant)	**>8.0 **(resistant)
CS^b^	**120.0 **(resistant)	**120.0 **(resistant)
CLA^b^	**0.5 **(susceptible)	**0.5 **(susceptible)
AZ^b^	**1 **(susceptible)	**1 **(susceptible)
AMK^b^	**8.0 ** (resistant)	**4.0 ** (susceptible)^c^
MOX^b^	**>1.0 **(resistant)	**1.0 **(resistant)^ c^
LZ^b^	**2.0 **(resistant)	**0.5 **(susceptible)^ c^
RBT^b^	**>2.0 **(resistant)	**0.25 **(susceptible)^ c^

^a^Drug resistance was assayed using indirect proportion method on Middlebrook 7H11. ^b^MICs were determined by the resazurin assay. ^c^Resitance values lower than those of the *M. avium *wild type strain.
